# PCA-derived respiratory motion surrogates from X-ray angiograms for percutaneous coronary interventions

**DOI:** 10.1007/s11548-015-1185-2

**Published:** 2015-04-07

**Authors:** Hua Ma, Gerardo Dibildox, Carl Schultz, Evelyn Regar, Theo van Walsum

**Affiliations:** 1 Biomedical Imaging Group Rotterdam, Departments of Radiology and Medical Informatics, Erasmus MC, Rotterdam, The Netherlands; 2 Department of Cardiology, Royal Perth Hospital, Perth, Australia; 3 Department of Cardiology, Erasmus MC, Rotterdam, The Netherlands

**Keywords:** Respiratory motion, X-ray angiograms, Principal component analysis, Percutaneous coronary intervention

## Abstract

**Purpose:**

Intraoperative coronary motion modeling with motion surrogates enables prospective motion prediction in X-ray angiograms (XA) for percutaneous coronary interventions. The motion of coronary arteries is mainly affected by patients breathing and heartbeat. Purpose of our work is therefore to extract coronary motion surrogates that are related to respiratory and cardiac motion. In particular, we focus on respiratory motion surrogates extraction in this paper.

**Methods:**

We propose a fast automatic method for extracting patient-specific respiratory motion surrogate from cardiac XA. The method starts with an image preprocessing step to remove all tubular and curvilinear structures from XA images, such as vessels and guiding catheters, followed by principal component analysis on pixel intensities. The respiratory motion surrogate of an XA image is then obtained by projecting its vessel-removed image onto the first principal component.

**Results:**

This breathing motion surrogate was demonstrated to get high correlation with ground truth diaphragm motion (correlation coefficient over 0.9 on average). In comparison with other related methods, the method we developed did not show significant difference ($p>0.05$), but did improve robustness and run faster on monoplane and biplane data in retrospective and prospective scenarios.

**Conclusions:**

we developed and evaluated a method in extraction of respiratory motion surrogate from interventional X-ray images that is easy to implement and runs in real time and thus allows extracting respiratory motion surrogates during interventions.

## Introduction

Percutaneous coronary intervention (PCI) is a minimally invasive procedure for treating patients with advanced coronary artery disease. With this technique, a catheter system is introduced into patients’ circulation system through their femoral or radial artery under local anesthesia. A preshaped guiding catheter is positioned into the ostium of the coronary artery. Through this catheter, a guide wire serving to deploy devices, such as balloon catheters and stents, is advanced into the branches of the artery. Once a stenosis is targeted, the balloon is deployed at the lesion site to fix the vessel blockage [1].

PCI is normally performed in a catheterization laboratory under the guidance of X-ray angiography (XA) that coronary arteries are opacified with contrast agent. However, due to contrast agent toxicity, its injection times are limited, such that guide wire and device advancement into the target lesion is performed under “vessel-free” images. In this situation, interventional cardiologists have to mentally reconstruct the position of coronary arteries and stenosis based on previous images, which increases the risk of failure for the procedures, especially in difficult cases.

To address this problem, Shechter et al. proposed to model the coronary motion with surrogate signals from contrast-enhanced images, and hence, the guidance in “vessel-free” images becomes feasible by prospective motion correction with such a model [3]. As the motion of coronary arteries is mainly affected by patient’s breathing motion and cardiac heart beat, it is reasonable to model the coronary motion with surrogates which are correlated with patients’ respiratory and cardiac motion. With such an aim, we have been focusing on respiratory-induced coronary motion modeling. Therefore, the purpose of our study was to develop and evaluate a method for fast and robust extraction of respiratory motion surrogates from X-ray angiograms for PCI.

Related works on respiratory motion surrogates have been reported. Signals from external apparatus, such as navigators or bellows, have been used in many studies for respiratory motion modeling [2]. Usage of image-based surrogates have also been investigated. One commonly used surrogate is diaphragm superio-inferior (SI) motion [3–5]. This is extracted by drawing a rectangular ROI on diaphragm border followed by manual tracking the diaphragm or automatic calculating the 1D translation. These methods involve human interaction to draw an ROI and hence not entirely automatic. Automatic diaphragm detection and tracking were reported in [6, 7]. These methods use morphological operation to preprocess XA images followed by a second-order curve fitting to the diaphragm border. Studies on other respiratory-related objects, e.g., coronary sinus catheter and tracheal bifurcation, can be seen in [4, 8]. These methods require specific objects being present in images, which is not always the case in XA images for PCI. Dimension reduction techniques have been used for studying respiratory motion as well. In [9], an automatic method based on principal component analysis (PCA) was designed for retrospective motion gating. This method first creates a mask using Hessian-based vesselness filtering and analyzes pixels inside the mask with PCA technique. In another study [10], hierarchical manifolding learning was used to find correlation between image regions and respiratory motion.

In this work, we developed a real-time, PCA-based method for extracting a respiratory surrogate from coronary XA sequences. Our contributions are threefold: First, we develop a method that is simple to implement and runs in real time on common PC hardware; second, we evaluate the method on several clinical datasets, comparing the results with manual ground truth and existing methods; third, we assess the usability of the method on monoplane and biplane image data in both retrospective and prospective scenarios.

## Methods

Coronary motion analysis in frames of XA sequence is complicated by the existence of both respiratory and cardiac motion in images. Therefore, respiratory motion surrogate extraction could possibly benefit from elimination of the objects representing cardiac motion from XA images. In this situation, respiratory motion becomes the major source of intensity change in XA sequence and could be analyzed with methods having source decomposition capability, such as principle component analysis.

To give an overview, our proposed method consists of two major steps. First, images are downsampled and processed with morphological closing to remove coronary arteries, guiding catheters, etc. Next, pixel intensity changes in the “vessel-removed” images are analyzed with principal component analysis to extract respiratory motion information. Each of the steps is explained in more detail in the next sections.

### Preprocessing of XA images

First, each frame of the sequence is downsampled. Depending on the original image size, the downsampling factor is chosen to be 4 if original size is $512\times 512$ or $600\times 600$, or 8 if previous size is $1024\times 1024$. This operation converts the original frame to an image of size $128\times 128$ or $150\times 150$, which already allows fast processing in later steps and still preserves enough original information that we need for subsequent analysis.

Next, as we are interested in respiratory motion only, we remove structures that show cardiac motion. To this end, similar to [6], a morphological closing is applied to the downsampled image with a circular structuring element in order to remove any tubular and curvilinear structures, such as coronary arteries, guiding catheters, guide wires and stitches. The size of the structuring element is chosen based on the maximal diameter of coronary arteries and guiding catheters. Dodge reported that the lumen diameter of the left main artery measures $4.5\pm 0.5$ mm [11]. In another study using transthoracic echocardiography [12], the average wall thickness of left anterior descending artery was $1.1\pm 0.2$ mm and its external elastic membrane diameter is $4.5\pm 0.9$ mm. Having a maximum coronary diameter of 5–7 mm and a maximum magnification of 1.5, we use a structuring element of around 11 mm in diameter (roughly 7–8 pixels in radius in the downsampled images) to remove the curvilinear structures. This size is shown to be adequate and guarantee a complete removal of vessels and guiding catheters from our images.

**Fig. 1:**
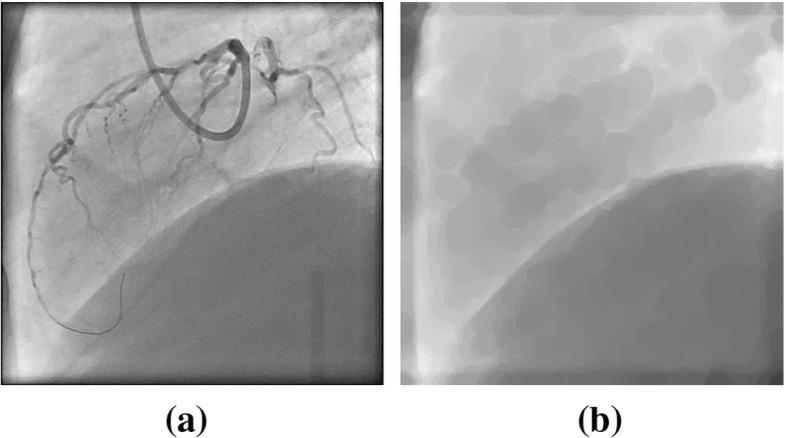
Morphological closing operation on an XA image. **a** Original XA. **b** Image processed with morphological closing: guiding catheter and coronary arteries are removed

With the mentioned operations, objects representing cardiac motion are effectively removed from downsampled XA images, while the diaphragm border and lung tissues still remain. Morphological closing might cause circular artifacts which, however, have lower contrast than the arteries, which is sufficient to prevent the subsequent analysis from being “contaminated” by cardiac motion. An example morphological-closed image is showed in Fig. 1.

### Principal component analysis

Principal component analysis (PCA) is typically used for dimension reduction. It transforms a multivariate dataset to a new orthogonal coordinate system such that most variance of this dataset could be represented by a few coordinates. Hence, reducing its dimension is normally achieved by preserving only a few coordinates in the new coordinate system without losing much information [14].

Similar to [9], we first use the PCA technique on morphological-closed images to obtain principal components for each sequence. Representing a frame of an XA sequence with an *n* $\times$ *n* matrix, we concatenate each pixel in such matrix into a single column vector $\mathbf x ^i$, whose size is *D* $\times$ 1, where $\textit{D} = \textit{n}^2$. Thus, an XA sequence consisting of *N* frames is represented as a $\textit{D}\times \textit{N}$ matrix $\textit{X} = [\mathbf x ^1,\ldots ,\mathbf x ^\textit{N}]$. We then center *X* to obtain a zero mean matrix. Without losing generality, we still write the zero mean matrix as *X*. Seeking the principal components of *X* is equivalent to computing the eigenvectors of covariance matrix $\textit{X}\textit{X}^T$, which is a $\textit{D}\times \textit{D}$ matrix. As *D* is usually a large number and in our case $\textit{D}>>\textit{N}$, we adopt the approach from [14] to apply eigen analysis to the $\textit{N}\times \textit{N}$ matrix $\textit{X}^T\textit{X}$. Then we have

1 $$\begin{aligned} E = X\tilde{E}\Lambda ^{-1} , \end{aligned}$$

where *E* is the $\textit{D}\times \textit{N}$ matrix of eigenvectors of $\textit{X}\textit{X}^T$, $\tilde{\textit{E}}$ is the $\textit{N}\times \textit{N}$ matrix of eigenvectors, and $\Lambda$ is the $\textit{N}\times \textit{N}$ diagonal matrix of eigenvalues of $\textit{X}^T\textit{X}$. With this approach, we benefit computation efficiency from computing the eigenvectors of a smaller matrix $\textit{X}^T\textit{X}$. Next, we project the XA sequence on the first principal component $\mathbf e _1$ by computing

2 $$\begin{aligned} \mathbf p = X^T\mathbf e _1 , \end{aligned}$$

where $\mathbf e _1$ is the first column of *E* representing the direction of the largest variance and **p** is a $\textit{N}\times 1$ projection vector. So each frame in such sequence is represented by an element in vector **p**. The assumption underlying our approach is that respiratory motion is the major source of variation in these sequences where cardiac motion is eliminated. Therefore, we use **p** as our breathing surrogate.

## Experiments

### Image data

For our experiments, we used anonymized imaging data that were acquired from Department of Cardiology at Erasmus MC (University Medical Center Rotterdam) in Rotterdam, the Netherlands. XA images of eight patients who underwent a PCI procedure that were acquired with Siemens AXIOM-Artis biplane system were analyzed. The frame rate of all sequences is 15 frames per second. The number of frames per series ranges from 55 to 244, corresponding to 3.7–16.3 s of imaging time. All eight patients have in total 1898 frames. From our image data, five are $512\times 512$ pixels, two are $600\times 600$, and one is $1024\times 1024$, with pixel size $0.216\times 0.216$ or $0.279\times 0.279$, $0.184\times 0.184$ and $0.139\times 0.139$ $\hbox {mm}^2$. The diaphragm can clearly be seen in seven patients in both images of the biplane data, whereas the diaphragm border is not visible in the other one. In that case, some lung tissues motion can still be observed and served as the main indicator of respiratory motion. Contrast agent injection and fading during imaging can be seen in all sequences.

### Ground truth data

**Fig. 2:**
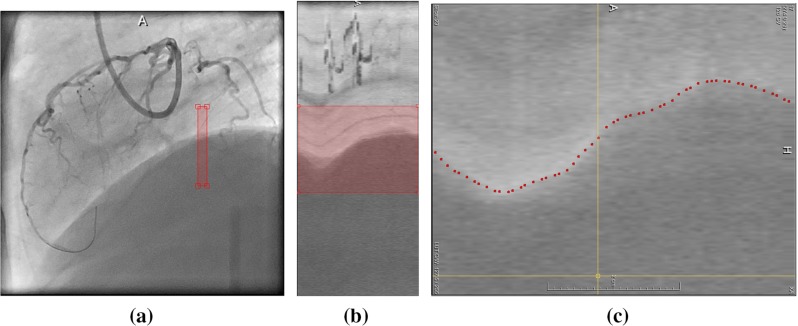
Ground truth data. **a** Drawing a rectangular ROI on diaphragm border. **b** Sagittal view of ROI. **c** Manual labeling of diaphragm border

Ideally, ground truth should be a direct indicator of respiratory motion. We first manually selected a rectangular ROI in original XA sequence on diaphragm border or where there is lung tissue motion, see Fig. 2a. Stacking all frames into a image volume and inspecting the “sagittal” view of the ROI, we observed a profile representing the change of diaphragm position, see Fig. 2b.

Manual labeling diaphragm or lung tissue’s motion track was subsequently done on the sagittal view of ROI image (Fig. 2c). This labeling was performed such that there is only one marker in each frame. This labeling step resulted in a vector of real numbers representing the diaphragm position in the ROI over time and it served as the ground truth data in our study.

### Retrospective evaluation

For retrospective evaluation, we used all frames of an XA sequence to compute the first principal component. The resultant projection vector **p** represents respiratory motion of such sequence and was compared with the previously mentioned ground truth diaphragm labeling. This comparison was quantified by calculating the correlation coefficient between projection vector **p** and ground truth vector. In order to gain insight of the usability of our method on different system, the retrospective analysis was tested with both monoplane and biplane data. For biplane data, we combined information from both planes by simply using the concatenated matrix $$\textit{X}=\bigl ({\begin{matrix}X_A \\ X_B\end{matrix}} \bigr )$$ in the same approach we have described in section “Principal component analysis”. By doing so, we could calculate one single projection vector **p** for both planes.

In addition, we compared the performance of our method, called *Vessel Removed* in later sections, with a recently published method that uses a masked-PCA approach [9]. Masked-PCA technique was designed for retrospective cardiac and respiratory motion gating on interventional cardiac X-ray images. In order to extract respiratory motion surrogate with this method, we slightly changed its implementation by directly using the projection vector on the first principal component without filtering it. We call it *With Mask* method in subsequent sections. We also investigated other possible variations of PCA-based methods, e.g., running PCA on the downsampled images without morphological closing (called *Downsampled Image*), and running PCA with an inverted mask of the mask created in [9] (called *Inverted Mask*). In all cases, the correlation coefficient of the resulting respiratory motion surrogate and the ground truth was calculated to quantify the performance on respiratory motion extraction.

### Prospective evaluation

We also evaluated whether the motion surrogate derived from our method could be used for prospective respiratory-induced coronary motion modeling in PCI. In this experiment, we only utilized a part of the sequence to build the PCA-derived model and used it to make predictions on subsequent frames. Considering a scenario in coronary motion modeling, we would build a model based on frames with contrast agent and use it to improve alignment of preoperative data onto XA on frames where contrast agent has been flushed away. This makes it reasonable to choose frames before contrast agent starts fading for the PCA learning phase and use frames without contrast for the prediction phase. Similar to retrospective experiment, we used the correlation coefficient to quantitatively evaluate the results with monoplane and biplane data and also compared with performances of other previously mentioned methods.

All experiments were implemented in MATLAB 2013b on an Intel Core2 2.66 GHz computer with 4 GB RAM running Windows. Computation time for each patient was recorded in all experiments.

## Results

### Retrospective analysis

**Fig. 3:**
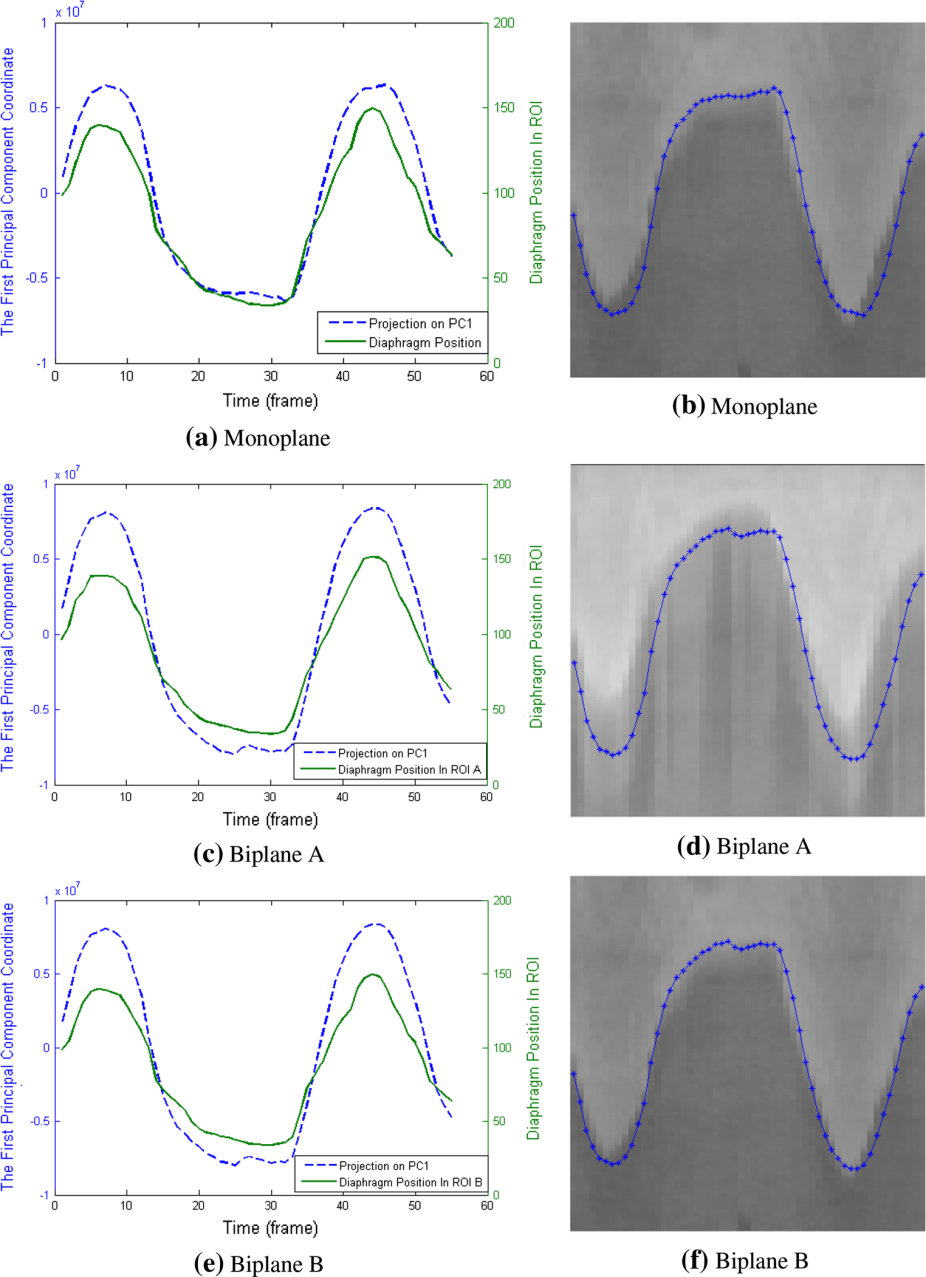
Retrospective projection on the first principal component for one patient, in comparison with diaphragm position in ROI image. **a**, **b** PCA was done on one sequence of the biplane data; **c**–**f** principal components were derived from the concatenated sequence of both planes. **c**, **d** show the projection in comparison with plane A; **e**, **f** illustrate the comparison with plane B

Example results of experiments on monoplane and biplane data from one patient are shown in Fig. 3. Figure 3a, c, e presents the comparison of projection vector **p** and ground truth diaphragm position. These figures show a high correlation between the two vectors. Linearly rescaling **p** to the range of ground truth data and overlaying it onto the ROI image provides another way to evaluate their correlation qualitatively. Figure 3b, d, f reveals a good agreement between **p** and diaphragm border.

**Fig. 4:**
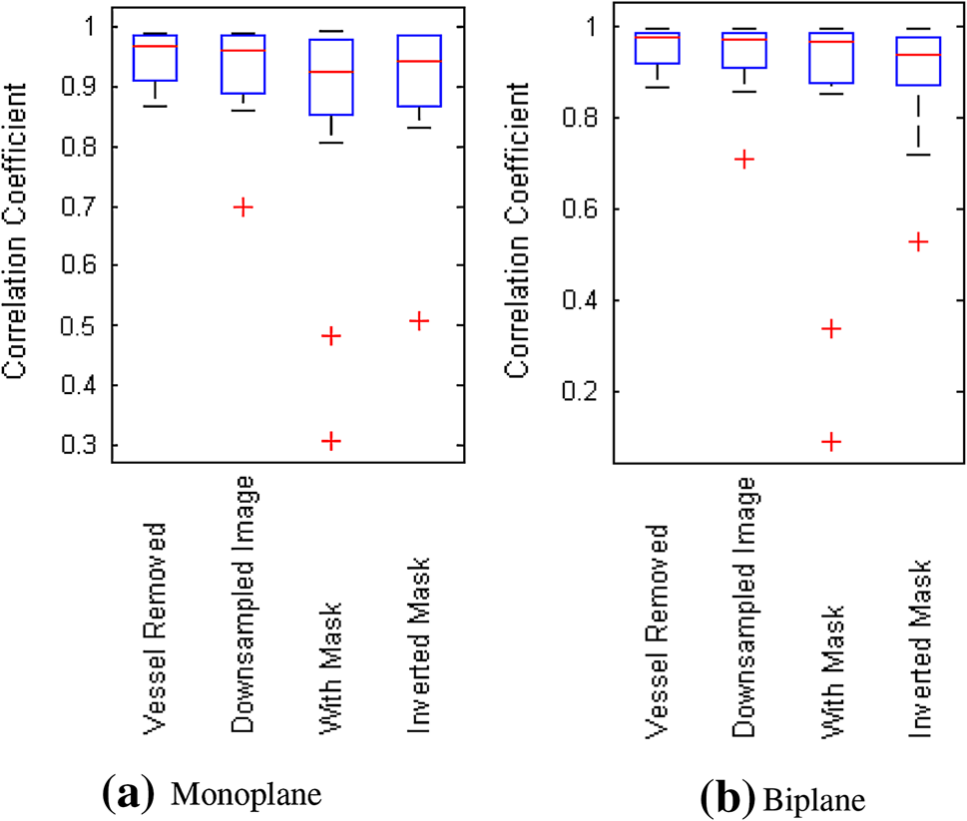
*Boxplot* of correlation coefficients calculated using various methods on monoplane and biplane data for retrospective evaluation

Table 1 provides quantitative measure results on correlation coefficients. The average correlation coefficient was calculated over all sequences for the various methods. From the table, it can be seen that all methods give high correlation coefficient (over 0.85, close to 1). The *Vessel Removed* method has slightly higher average correlation and lower standard deviation than other methods. For the patient whose XA sequences contain no diaphragm border, the correlation coefficients for *Vessel Removed* method are also high for the monoplane (0.89 and 0.88) and biplane data (0.88 and 0.95). Boxplots in Fig. 4 illustrate similar observations: The majority of correlation coefficients are over 0.8 for all methods; non-mask-based methods (*vessel-removed* and *Downsampled Image*) slightly outperform other methods.

**Table 1:** Average correlation coefficient of projection vector **p** and diaphragm positions for various methods for retrospective evaluation

Methods	Monoplane	Biplane
	(mean $\pm$ std)	(mean $\pm$ std)
Vessel Removed	0.9490 $\pm$ 0.0446	0.9529 $\pm$ 0.0424
Downsampled Image	0.9330 $\pm$ 0.0754	0.9357 $\pm$ 0.0742
With Mask	0.8637 $\pm$ 0.1883	0.8552 $\pm$ 0.2503
Inverted Mask	0.9032 $\pm$ 0.1156	0.9007 $\pm$ 0.1197

To investigate whether there is a statistically significant difference between the performance of these methods on retrospective respiratory motion surrogates extraction, we used a one-tailed Wilcoxon rank-sum test to check the correlation coefficients, as their distribution is not necessarily a normal distribution and most of values are close to 1. Result (see Table 2) shows that the *p* values for monoplane experiments range from 0.09 to 0.55 (in upper triangle); *p* values for biplane experiments range from 0.11 to 0.42 (in lower triangle). The result means these methods have similar performance on retrospective task in terms of statistical significance.

**Table 2:** Statistical significance between various methods for retrospective evaluation (*p* values). The numbers in the upper and lower triangle in the table show the results of monoplane and biplane cases respectively

	Vessel Removed	Downsampled Image	With Mask	Inverted Mask
Vessel Removed	$\times$	0.28	0.15	0.09
Downsampled Image	0.31	$\times$	0.24	0.22
With Mask	0.23	0.42	$\times$	0.55
Inverted Mask	0.11	0.24	0.36	$\times$

**Fig. 5:**
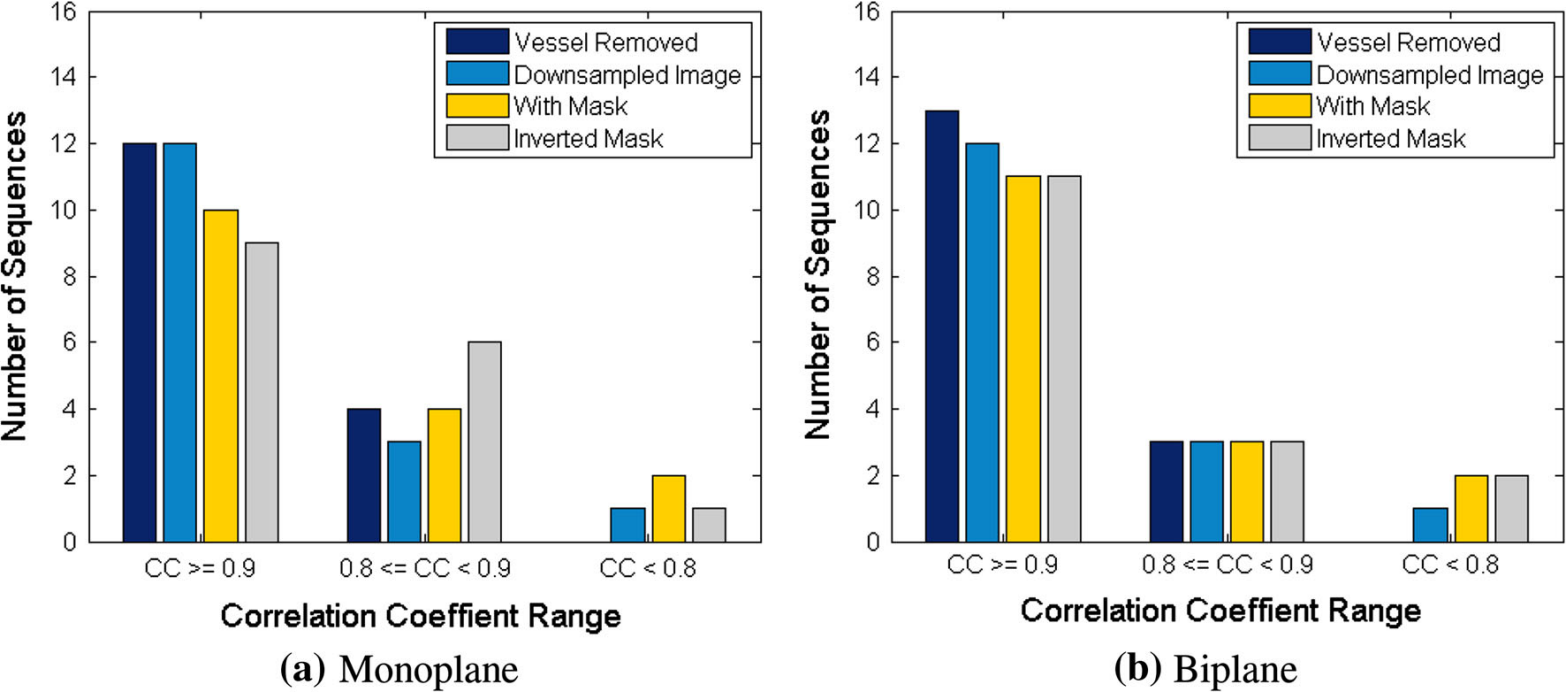
Frequency distribution of correlation coefficients for various methods in retrospective analysis

Figure 5 illustrates the frequency distribution of correlation coefficients for various methods in our retrospective analysis. In both the monoplane and biplane experiments, it can be observed that *Vessel Removed* method has the most number of correlation coefficients over 0.9 and no correlation coefficients lower than 0.8, which outperforms all other methods. This observation suggests that the *Vessel Removed* method is more robust than the other approaches.

Table 3 compares the average per-frame computation time that is needed to compute the projection vector **p**. This includes the time for image preprocessing, building statistical model through PCA and making projection on the first principal component. The comparison reveals the advantage of non-mask-based methods to mask-based method that the computation time they need is much shorter, which is favored for real clinical workflow.

**Table 3:** Average per-frame computation time for various methods for retrospective evaluation (milliseconds)

Methods	Monoplane (ms)	Biplane (ms)
Vessel Removed	17.2	34.4
Downsampled Image	8.0	15.5
With Mask	821.6	2227.8
Inverted Mask	854.7	2920.0

### Prospective analysis

**Fig. 6:**
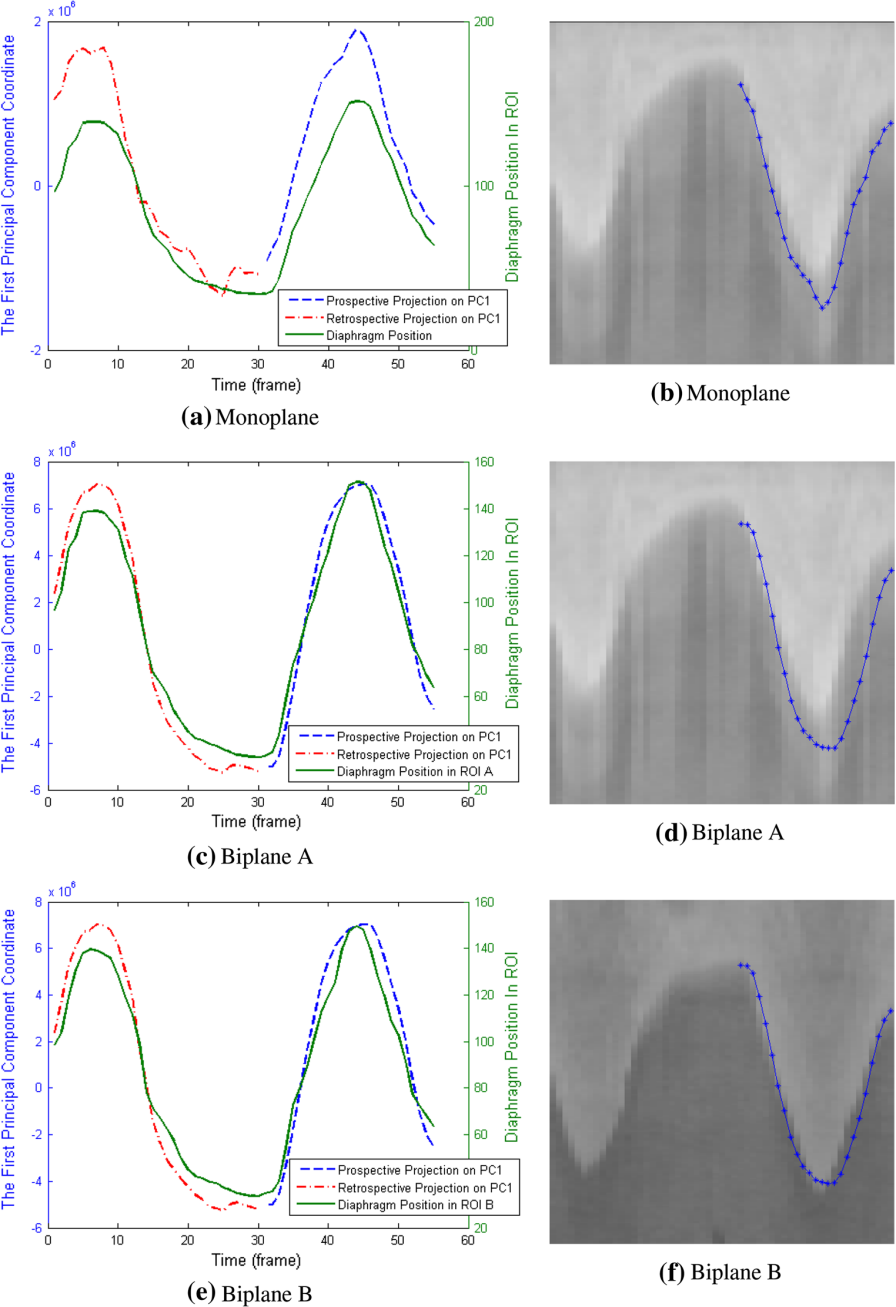
Prospective projection on the first principal component for one patient, in comparison with diaphragm position in ROI image. **a**, **b** PCA was implemented on monoplane data; **c**–**f** principal components were derived from the concatenated sequence of biplane. **c**, **d** show the projection in comparison with plane A; **e**, **f** illustrate the comparison with plane B

Example results of the prospective analysis for the same patient as in Fig. 3 are shown in Fig. 6. Figure 6a, c, e present retrospective projection for frames used for learning the statistical model and prospective projection for frames excluded from the learning phase. It can be seen that the prospective projection vector **p** still maintains good correlations with ground truth diaphragm position.

**Fig. 7:**
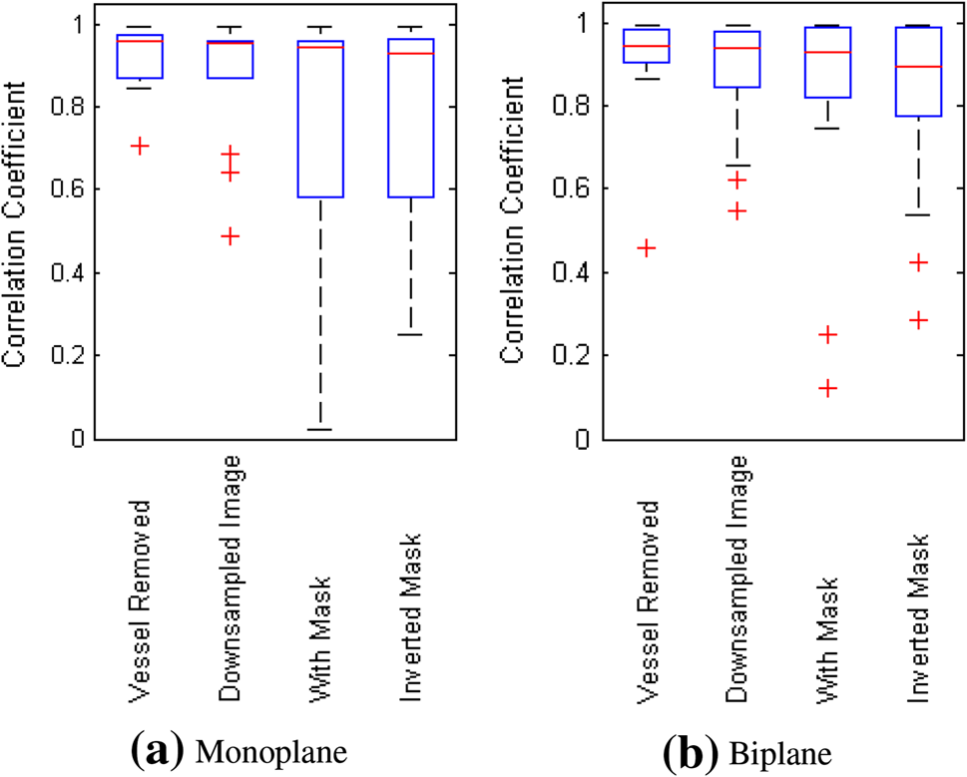
*Boxplot* of correlation coefficients calculated using various methods on monoplane and biplane data for prospective evaluation

Correlation coefficients are shown in Table 4. In general, these numbers are lower than those in the retrospective experiments, while *Vessel Removed* method still maintains a high average correlation coefficient over 0.9. Its standard deviation is also lower than other methods. For the patient whose diaphragm cannot be seen in the XA sequences, the correlation coefficients for *Vessel Removed* method remain good for one of sequences in the monoplane data (0.96 and 0.70) and both sequences in the biplane data (0.91 and 0.87). Boxplots in Fig. 7 show that the medians of all methods are quite close to each other, but *Vessel Removed* method has fewer correlation coefficients lower than the median value compared to other methods. We also used Wilcoxon rank-sum test to check the statistical significance, and the results are shown in Table 5. No significant difference is found among these methods ($p>0.14$), which means their performance are similar to each other in terms of statistical significance.

**Table 4:** Average correlation coefficients of the prospective projection vector and diaphragm positions for various methods

Methods	Monoplane	Biplane
	(mean $\pm$ std)	(mean $\pm$ std)
Vessel Removed	0.9197 $\pm$ 0.0733	0.9128 $\pm$ 0.1239
Downsampled Image	0.8823 $\pm$ 0.1418	0.8815 $\pm$ 0.1413
With Mask	0.7548 $\pm$ 0.3291	0.8282 $\pm$ 0.2540
Inverted Mask	0.7772 $\pm$ 0.2649	0.8201 $\pm$ 0.2117

**Table 5:** Statistical significance between various methods in prospective evaluation (*p* values). The numbers in the upper and lower triangle in the table show the results of monoplane and biplane cases respectively

	Vessel Removed	Downsampled Image	With Mask	Inverted Mask
Vessel Removed	$\times$	0.31	0.15	0.17
Downsampled Image	0.52	$\times$	0.18	0.17
With Mask	0.31	0.36	$\times$	0.57
Inverted Mask	0.18	0.19	0.39	$\times$

**Fig. 8:**
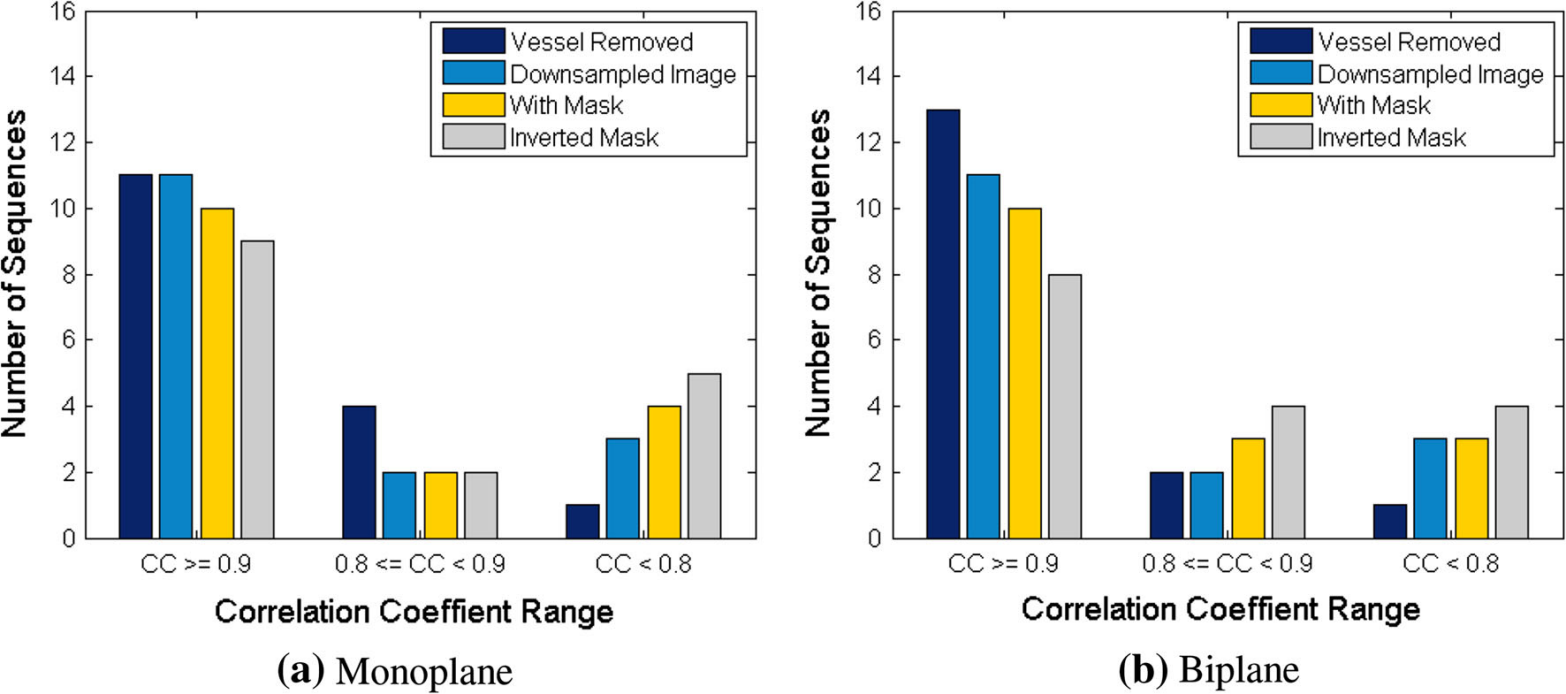
Frequency distribution of correlation coefficients for various methods in prospective analysis

Frequency distribution of correlation coefficients in Fig. 8 reveals that for both monoplane and biplane experiments, more lower value correlation coefficients appear for all methods compared to retrospective analysis. It is also clear that *Vessel Removed* method has the most high correlation coefficients (CC $\ge$ 0.9) and the least lower correlation coefficients (CC $<$ 0.8) among all methods.

Average per-frame prediction time for various methods is listed in Table 6. The prediction time includes the time for image preprocessing and computing projection on the first principal component. It is obvious that *Downsampled Image* method has the shortest prediction time, while *Vessel Removed* method is also quite fast. Mask-based methods are slower than non-mask-based methods, especially when they were run on biplane data.

**Table 6:** Average per-frame prediction time for various methods (milliseconds)

Methods	Monoplane (ms)	Biplane (ms)
Vessel Removed	9.6	20.0
Downsampled Image	1.6	3.8
With Mask	16.3	282.6
Inverted Mask	31.0	792.4

## Discussion

We developed an automatic method to extract patient-specific respiratory motion surrogate from cardiac interventional X-ray angiograms using principal component analysis. The method was evaluated on monoplane and biplane data in both retrospective and prospective manner.

Our experiments demonstrated that *Vessel Removed* method is able to extract breathing information having high correlation with the ground truth diaphragm or lung tissue motion. The average correlation coefficient is higher than those for other related methods in our experiments. *Vessel Removed* method is also more robust than the other three methods giving that more correlation coefficients for the method are over 0.9 and less are below 0.8.

It is also observed that the difference between the mentioned methods in this paper is yet not so profound that no statistically significant difference on the correlation coefficients was found. This might be due to the choice of similarity metric. Correlation coefficient is although one of the common ways to measure the similarity of two time series signals, there are other measures which could potentially improve the difference between these algorithms, such as distance correlation [13].

The limited number of patients (only eight) may also be a reason for the lack of statistical significance. In the future, we will evaluate the method on a much larger set of patients. Despite the lack of statistical insignificance, the *Vessel Removed* method performs at least as good as the other three approaches.

From the aspect of computation efficiency, the time that *Vessel Removed* method needs for building the statistical model and making prediction on our computer are less than 67 ms, corresponding to the 15-Hz imaging rate of our data, whereas mask-based methods need longer time to accomplish the same task. This means that *Vessel Removed* method could run in real time.

Image-based respiratory motion surrogates in interventional X-ray angiograms have been studied previously [3–5]. These works either need manually putting a rectangular ROI or require specific object being present in images. The method we have presented is fully automatic and more robust to various image content. The diaphragm is not necessarily required to be present as long as there is sufficient breathing motion observed, which is true in most of the cases in PCI procedures since the lung tissue is usually seen in the background.

The application of dimensional reduction techniques in extraction of respiratory motion information was seen in [9, 10]. [10] presented one example case, and the method in [9] was originally designed for retrospective gating. In comparison with these works, the method we have developed is simpler and needs no vessel extraction from images thus is also faster. In addition, we have evaluated the usability of our method on monoplane and biplane data in retrospective and prospective manner and achieved good correlation in both tasks.

**Fig. 9:**
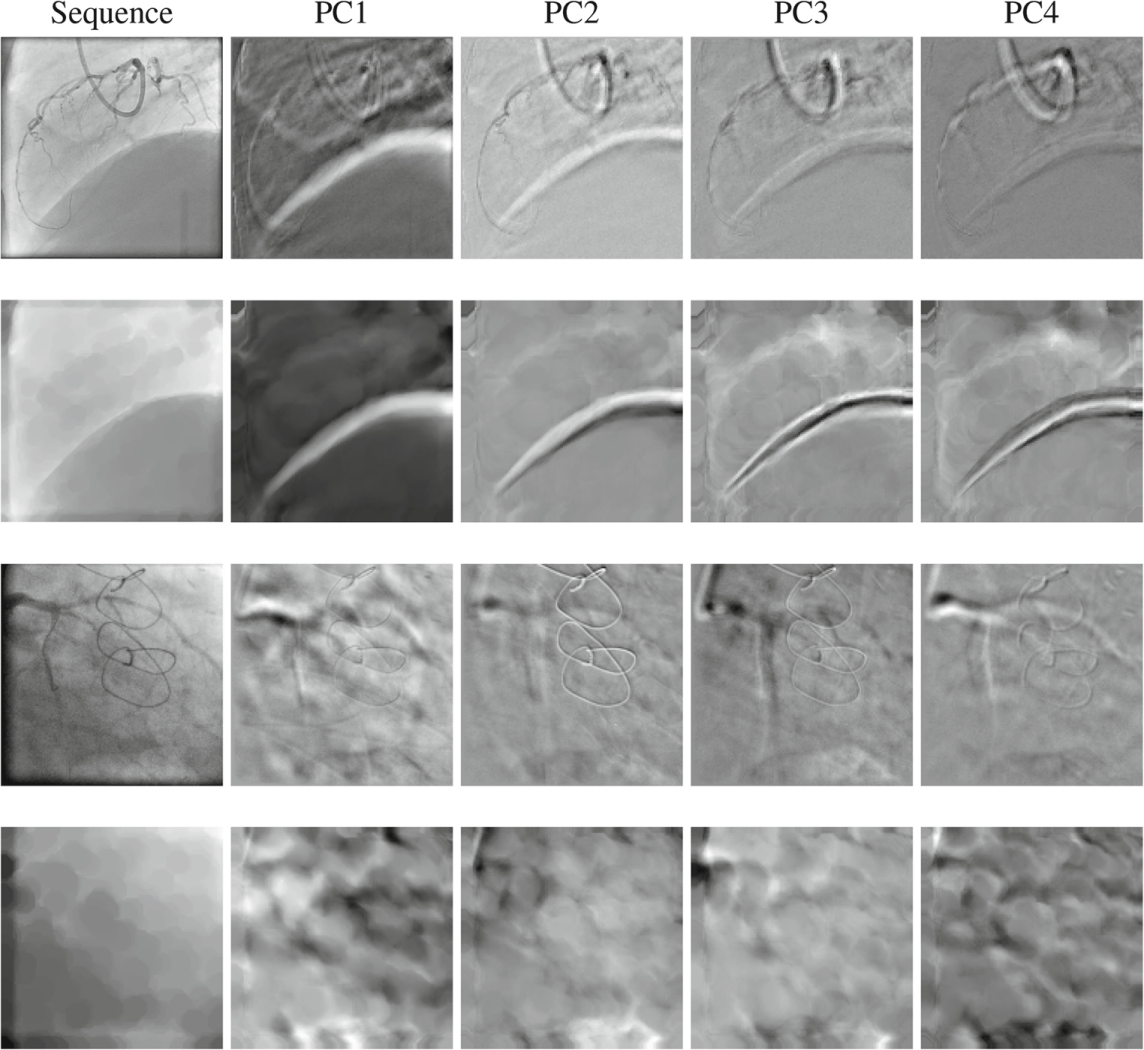
The first four principal components (eigenimages) of images with and without diaphragm being present. The first two rows of images have diaphragm and the last two rows do not. The first and third row show the original images and their eigenimages, and the second and fourth row are the same images in the first and third row after morphological closing operation

Observations on principal components of XA images would help understanding the mechanism of our proposed method. The first four principal component images, called “eigenimages,” of two example sequences with and without diaphragm are shown in Fig. 9. It is obvious that in both cases, the cardiac motion pattern is still present in eigenimages of the original images, but significantly suppressed in those of the morphological-closed images. Also in vessel-removed images, for the case with a diaphragm, the diaphragm border is enhanced in the eigenimages showing a white or dark stripe, while in the sequence where diaphragm is not present, white and dark pixels represent background lung tissues. It can also be observed that the first eigenimage contains strong respiratory motion signals which makes it reasonable of projecting XA sequence on the first principal component to obtain breathing motion feature.

In prospective surrogate extraction, the frames that are needed for building the statistical model are required to cover the maximal range of respiratory motion; therefore, our proposed method might be limited for different breathing patterns. The method we developed is also only applied to one fixed view angle. Detector position changes during interventions require model rebuilding, which having been seen to be fast in our experiments.

The method we developed could directly be used for patient-specific coronary motion modeling. Its short modeling and prospective extraction time enables the possibility of running in real time and being used during interventions. Due to the robustness of the method to different image contents, it could also be potentially used for extraction of respiratory motion surrogate for other types of interventions using different imaging modalities.

In the future, we will extend the study with more patients’ data. We will also investigate on cardiac motion surrogates extraction from XA sequences with similar framework and adapting the current approach to varying view angles.

## Conclusion

We have presented a fast automatic method that can be used to retrospectively and prospectively extract patient-specific respiratory motion surrogate from cardiac XA sequences. Our experiments demonstrate a high correlation coefficient with manual ground truth: Average correlation coefficients are over 0.9 in the retrospective and prospective evaluations. The method is easy to implement and runs in real time and thus allows to extract respiratory motion surrogates during interventions.
